# When Pulmonary Arterial Hypertension may be Associated with Portal Hypertension: A Case Report of Two Different Hepatic Disorders in One Patient with Pulmonary Hypertension

**DOI:** 10.2174/011573403X267162231011154808

**Published:** 2023-10-20

**Authors:** Ganna D. Radchenko, Yuriy M. Sirenko

**Affiliations:** 1 State Institution National Scientific Center “Institute of Cardiology, Clinical and Regenerative Medicine named after acad. M.D. Strazhesko” of National Academy of Medical Science, Kyiv, Ukraine

**Keywords:** Reno-splenic, pulmonary arterial hypertension, hepatic disorders, prognosis, portal hypertension, biopsy

## Abstract

**Background:**

Pulmonary arterial hypertension (PAH) is a rare complication of hepatic diseases with portal hypertension that, however, has a significant influence on prognosis. We present a mini-review of how to diagnose and treat it based on a clinical case.

**Case Presentation:**

In early childhood, a patient had portal hypertension associated with cavernous transformation of the portal vein. It was successfully treated by reno-splenic surgery. At the age of 20 years, this patient experienced increased dyspnea at minimal physical activity after the hepatic biopsy due to a hepatocellular adenoma. The examination in the specialized unit showed PAH, which was evaluated as associated with portal hypertension (PAH-PoH). The specific two-drug combination therapy was started with prominent improvement in patient’s state. Successful surgical tumor treatment was provided some months later. The practical and clinical approaches to the diagnosis and treatment of PAH-PoH are discussed. It was emphasized that not all patients with portal hypertension have pulmonary hypertension, which needs to be treated. A lot of evidence gaps exist in management of these patients.

**Conclusion:**

All patients, even with past history of portal hypertension, should be monitored closely and screened for PAH earlier, for better results of treatment.

## 1 INTRODUCTION

Pulmonary arterial hypertension (PAH) is a rare (orphan) progressive disease with a lethal outcome without appropriate treatment within 3-5 years. It is characterized by chronically elevated blood pressure in the pulmonary arteries, which, in turn, is caused by increased pulmonary artery resistance. The last ESC-2022 guidelines identify PAH as a mean pulmonary arterial pressure of > 20 mmHg at rest, pulmonary capillary wedge pressure (PCWP) of < 15 mmHg and pulmonary vascular resistance (PVR) >2 Wood units at right heart catheterization (RHC), that is the main diagnostic method for PAH confirmation [1].

The pathomorphology of PAH includes the small pulmonary artery remodeling due to media hypertrophy, plexiform lesion, and microthrombosis (thrombus *in situ*). The progression of these pulmonary artery changes leads to increase of the pulmonary blood pressure and right ventricle overload, along with decrease in the left ventricle filling [1, 2]. Some pathophysiological mechanisms for this remodeling were studied, and now we have a lot of information about the metabolism of the vasoconstrictors (endothelin-1, thromboxane, *etc*.) and expression of their receptors, along with prostacyclin, nitric oxide, and inflammation factor activity.

The pathogenic mechanisms of PAH are multifactorial and common for all PAH types (group 1), but the etiology and triggers could be different. If no underlying diseases were found, it could be classified as idiopathic. If genetic abnormalities, drug/toxin usage, or such diseases as connective tissue diseases, portal hypertension, human immunodeficiency virus, or congenital heart conditions are present, PAH is defined as ‘associated with’ [1, 2]. Hepatic diseases might be accompanied by pulmonary hypertension, but not always this hypertension could be classified as PAH. More often, the elevation of the pulmonary blood pressure is a consequence of the increased cardiac output due to portocaval anastomosis without a high PVR [3]. PAH could be identified very rare, but this diagnosis is associated with a poor prognosis for hepatic patients and bad results of their surgical treatment, including liver transplantation [4]. That is why it is very important to diagnose and treat PAH earlier.

We present an unusual clinical case report of the late diagnosis of PAH associated with portal hypertension in a patient who had two significant hepatic diseases at different ages. Portal hypertension was diagnosed in early childhood and treated by an artificial splenorenal shunt. But at adult age, the patient started to have clinical signs of PAH during the preparation for surgical treatment of the other hepatic disease. The patient gave us written permission to use his data in clinical studies and presentations.

## 2 CLINICAL CASE PRESENTATION

**Fig. (1):**
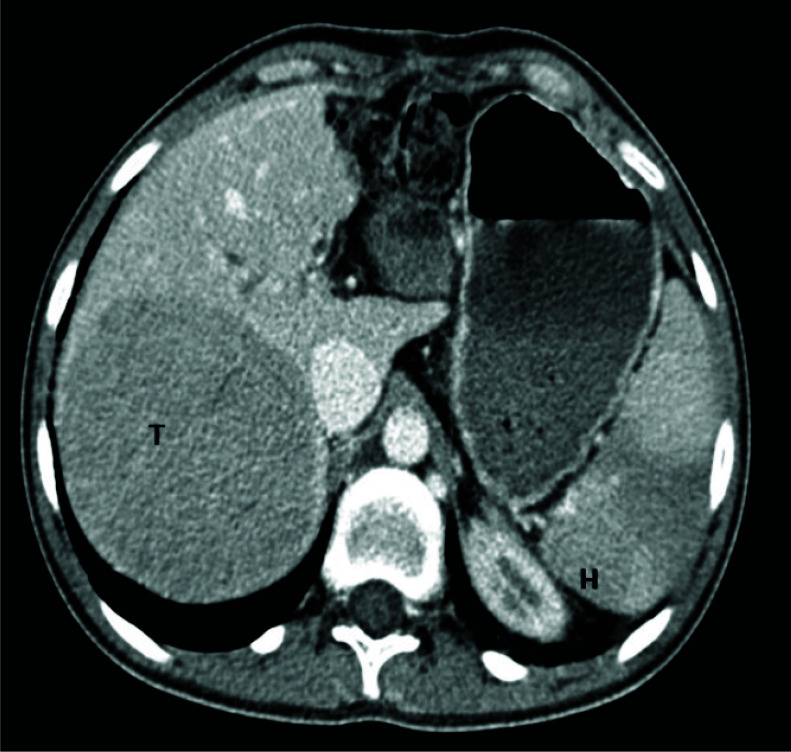
Computer tomography abdominal image: The large tumor in the right lobe of the liver, the hemangioma (H) in S VIII of the spleen.

**Fig. (2):**
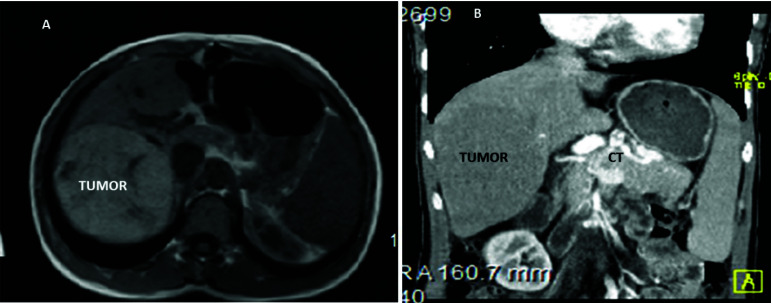
(**A**, **B**) Magnetic resonance abdominal image: Right lobe hepatic tumor (hepatocellular adenoma), obliteration and cavernous transformation (CT) of the portal vein, splenomegaly.

The 20-year-old patient had experienced increasing dyspnea with minimal physical activity after the hepatic biopsy procedure. In early childhood, he had portal hypertension associated with the cavernous transformation of the portal vein. Because of the repeating esophageal hemorrhage, sclerotherapy was provided three times, but it failed to stop the bleeding permanently. Splenomegaly and hypersplenism were diagnosed too. At age 4, splenorenal shunting was done with bleeding elimination and portal hypertension was completely cured. In the patient’s medical records, we did not find any data about signs of pulmonary hypertension at echocardiography (EchoCG). At 16 years of age, during the control abdominal ultrasound investigation, signs of the hepatic tumor were found in the right lobe. Computer tomography (CT) and magnetic resonance imaging (MRI) scans were provided, and the diagnosis of the benign tumor was confirmed. One year later, the patient was referred to the oncology clinic because of the tumor enlargement (Figs. **1** and **2**).

**Fig. (3):**
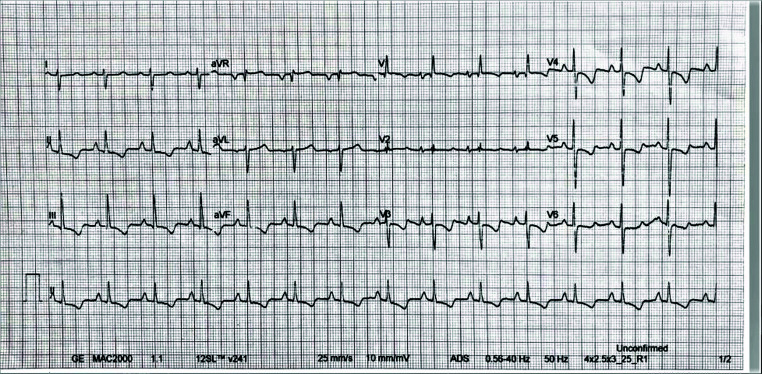
ECG: Sinus rhythm, the right bundle branch block, the right axis deviation and the right ventricle strain

**Fig. (4):**
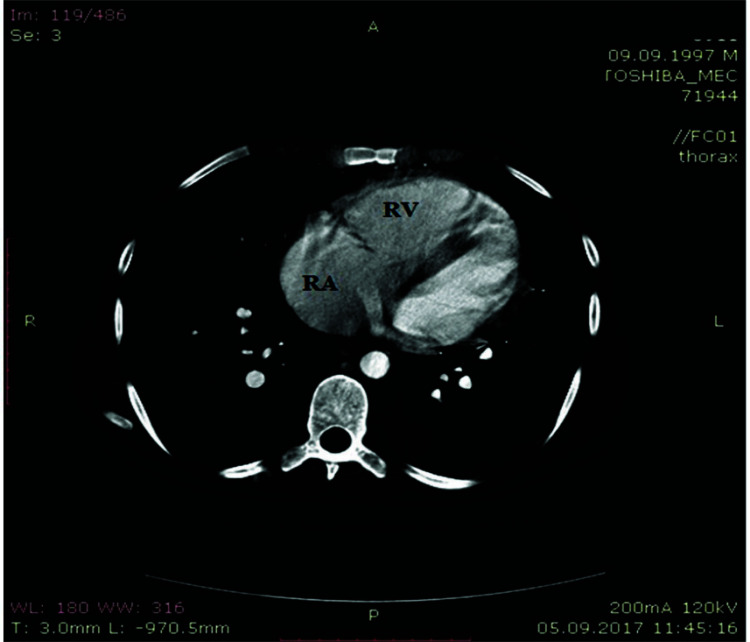
CT scan – the right heart enlargement.

In the oncology clinic, the hepatocellular adenoma was confirmed by tumor biopsy. The biopsy procedure was complicated by the abdominal bleeding (~ 2000 ml), the bleeding was interrupted by abdominal surgery only. The patient started to suffer from increasing dyspnea, being on infusion therapy and blood transfusions. On EchoCG, the high probability of pulmonary hypertension was established: The right heart enlargement, hydropericardium and the estimated systolic pulmonary blood pressure (SPBP) was 100 mmHg. The patient was moved to the secondary and pulmonary hypertension department in the State Institution National Scientific Center “Institute of Cardiology, Clinical and Regenerative Medicine named after acad. M.D. Strazhesko” of the National Academy of Medical Science. No cardiovascular disease history was found. The physical examination data were: office systolic/diastolic blood pressure - 103/68 mmHg, heart rate (HR) – 103 per min, breath rate at rest – 22 per min; SpO_2_ – 92%, right heart board enlargement, stress of the second tone over the pulmonary artery, tricuspid valve systolic murmur, WHO functional class IV. The electrocardiography data were: Sinus tachycardia, right axis deviation, right bundle branch block, and the right ventricle strain. (Fig. **3**). The young patient demonstrated poor functional capability at the 6-minute walk test (6MWT): the distance – 161 m, the Borg score – 6.0; SpO_2_ - 92% at baseline and 92% at the end of the test; HR - 102 per min at baseline and 125 per min at the end of the test. The EchoCG data were: the left atrium square – 18.5 cm^2^, the left atrium index - 13 ml/m^2^, the right atrium square - 28 cm^2^, the right atrium index - 65 ml/m^2^, the right ventricle long size – 8.7 cm, the right ventricle wide size – 5.2 cm, TAPSE – 16.3 mm, the pulmonary artery size - 4.0 cm, the estimated SPBP - 100 mmHg, the tricuspid regurgitation velocity - 4.8 m/s, the eccentric index in systole – 2.42, the eccentric index in diastole -1.92. The spirometry test was normal. We found no evidence of pulmonary artery thrombosis at the CT scan, just right heart enlargement (Fig. **4**). The blood analysis data were: WBC – 15.5 * 109/l, RBC – 3.9*1012/l, Hb – 100 g/l, NT-proBNP - 2115 pg/ml, bilirubin – 29 µmol/l, AST – 81 U/l, ALT – 123 U/l, ALP – 219 U/l, total protein – 60 g/l, ferritin – 22.4 ng/ml. RHC data demonstrated severe precapillary pulmonary hypertension: mean PAP - 59 mmHg, HR - 88 beat per min, right atrium pressure – 6 mmHg, PCWP – 6 mmHg, cardiac output – 3.33 l/min, cardiac index – 1.8 l/min/m^2^, PVR - 1273 dyn*s/cm^5^. The vaso-reactive test with iloprost was negative. PAH associated with portal hypertension was diagnosed based on the history of portal hypertension and the artificial reno-splenic shunt.

The administered therapy was: Sildenafil 20 mg tid, inhaled iloprost 5 µg 8 times per day, spironolactone 25 mg, iron polymaltose complex 100 mg, and torasemide 2.5 mg. The patient was discharged with an improving: 6MWT distance – 378 m, Borg score – 3.0; SpO2 97→95%; HR - 98 per min at baseline and 115 per min at the end of the test.

## 3 RESULTS

The RHC was repeated on therapy 3 months later. The mean PAP was 27 mmHg, and the right hepatic resection was performed successfully. The out-patient visit showed a significant enhancement in patient status on the specific therapy at the third month after surgery (table): No complaints, functional class I, breath rate at rest – 16 per min, office systolic/diastolic BP – 110/70 mmHg, HR – 68 per min, NT-proBNP - < 20 pg/ml, 6MWT distance – 518 m, Borg score – 1.0; SpO2 97→97%; 6MWT HR 82 per min at baseline and 110 per min at the end of the test. The EchoCG improvement was noted too: The left atrium square – 20 cm^2^, the left atrium index - 34 ml/m^2^, the right atrium square -20.2 cm^2^, the right atrium index – 38 ml/m^2^, the right ventricle long size – 8.5 cm, right ventricle wide size – 4.1cm, TAPSE - 21 mm, tricuspid regurgitation velocity – 4.1 m/s, the estimated SPBP – 73 mmHg, the eccentric index in systole -1.73, eccentric index in diastole – 1.24. Hematology and biochemical analysis data were normal. The patient successfully finished university and lives a full life on PAH therapy.

## 4 DISCUSSION

PAH in patients with portal hypertension is not frequently diagnosed – only 1–5% [1]. The pulmonary hypertension rate could be increased to 8.5% in patients directed to liver transplantation [2]. Mostly, PAH associated with portal hypertension (PAH-PoH) is diagnosed in women, with autoimmune liver diseases, and estrogen disorders. PAH-PoH is very occasional at the C virus hepatitis [4]. The PAH-PoH takes fourth place among PAH causes after the idiopathic PAH, the PAH associated with connective tissue diseases, or congenital heart diseases. It could reach 15% of all PAH group patients, with the growing number of cases as more extensive screening practices are provided [5, 6]. The deviations in screening practices for PAH in different countries could explain the large variety in the prevalence of PAH-PoH: In the USA and Spain, patients with severe liver diseases have to be screened for PAH just before liver transplantation, while in France it should be done at any time after the liver disease is diagnosed [7].

According to the current pulmonary hypertension guidelines, PAH-PoH criteria include: 1) clinical signs of portal hypertension (splenomegaly, portosystemic shunts, thrombocytopenia, portal vein abnormalities, esophageal varices, increased portal vein pressure during hemodynamic measurements) with or without liver disease; 2) the RHC data, including the mean PAP > 20 mmHg at rest, the mean PCWP < 15 mmHg, PVR > 2 Wu [1, 3, 8, 9]. The results of RHC are essential, because 30–50% of hepatic cirrhosis patients have high cardiac output and low systemic resistance, [10] that leads to the amplifying of pulmonary pressure. That is why, in some patients, pulmonary hypertension could be diagnosed. However, if the PVR is less than 2 Wu, this type of pulmonary hypertension should be classified as group 5 (due to the hyperdynamic circulation) and specific pulmonary hypertension therapy should not be prescribed.

The cause of PAH-PoH development is not well established, but the main mechanism is the pulmonary blood overflow (due to portosystemic shunts) in chronic liver disease. It could trigger the pulmonary vascular wall shear stress, and be the reason for the dysregulation of different vasoactive, proliferative, and angiogenic substances, which provoke small artery remodeling like in idiopathic PAH [11]. The constant portosystemic shunts, pro-inflammation factor circulation, endotoxins, and intestinal macrophages could maintain and aggravate the pulmonary artery damage [12]. Additionally, genetic factors might be crucial in PAH-PoH development too. The endothelin-1 upsurge, endothelin receptor dysregulation, the rise of thromboxane-B1, interleukin-6, and serotonin could activate pulmonary artery vasoconstriction too [13-17] There is some evidence about prostacyclin synthase deficient, nitric oxide, and prostaglandin I2 lowering in patients with portal hypertension [18].

PAH-PoH patient survival was compared with idiopathic PAH patients (non- vasoreactive): 88, 75, and 68% at 1-, 2- and 5 years [19]. The mortality risk of liver transplantation or liver surgery is increased in severe PAH-PoH. The 5-year survival rate was 14% in PAH-PoH patients without liver transplantation and specific PAH drug treatment *vs*. 45% without liver transplantation, but with specific PAH drug treatment, 25% with transplantation, but without PAH treatment, *vs.* 67% with transplantation and with specific PAH drug treatment [20]. Before liver transplantation, all patients should be screened for PAH, as PAH is a mortality risk factor. In the case of PAH-PoH diagnosis, they should be treated with specific therapy and achieve the target mean PAP < 35 mmHg and PVR < 400 dyn/s/сm^5^ [21, 22].

In some comparative studies, the PAH-PoH patients had a better hemodynamic profile and functional capacity but worse survival than patients with idiopathic PAH [23, 24].

There were at least two explanations for this. First, patients with PAH-PoH have a death associated with hepatic events in 25-33% of the cases, whereas idiopathic PAH patients have only 5% [19, 24]. The severity of hepatic disease (very often evaluated by the Child-Pugh Score) is linked with a poor prognosis if there is no pulmonary hypertension [19, 25]. For this reason, patients with PAH-PoH have an additional baseline mortality risk factor compared to patients with idiopathic PAH. Second, patients with PAH-PoH have a less aggressive specific treatment, the onset of which may be delayed quite often. In the REVEAL registry, the proportion of patients receiving vasodilatory therapy was lower at the time of enrolment and 90 days in the PAH-PoH group than in the idiopathic/heritable PAH group, [23], despite the evidence that PAH-PoH patients have a better prognosis if they are on the specific PAH treatment compared to PAH-PoH patients who are not on therapy [24]. The physician’s caution in the treatment prescription could be explained by: 1) the undervaluing of the patient’s condition (better baseline functional and hemodynamic status), 2) concerns about liver function worsening due to PAH drug hepatic adverse reactions, and 3) late detection of pulmonary hypertension (for example, in some countries patients are screened for PAH just prior to transplant or surgery only).

The diagnostic algorithm for pulmonary hypertension associated with portal hypertension is excellently summarized in Porres-Aguilar’s *et al*. review [9]. EchoCG must be done in all patients with portal hypertension and the clinical signs of pulmonary hypertension, and in all patients submitted to transplantation. At an estimated SPBP of less than 30 mmHg, the patient may be considered as “without pulmonary hypertension”. At an estimated SPBP between 30 and 50 mmHg, the patient may be asked to provide EchoCG within the next 6−12 months, or RHC could be provided. If the estimated SPBP is more than 50 mmHg, the RHC must be performed for the PAH diagnosis. If the mean PAP is less than 35 mmHg, liver transplantation/surgery is possible in the absence of any other contradictions. In cases where mean PAP is between 35 and 50 mmHg, PVR should be considered: at PVR < 250 dyn/s/cm^5^, liver transplantation/surgery is possible, at PVR > 250 dyn/s/cm^5^, specific PAH therapy must be initiated, and with a positive response, intervention is possible. Based on the American Association for the Study of Liver Disease and the Society of Transplantation guidelines, liver transplantation might be applicable if PVR could be lowered to <5 WU (400 dyn/s/cm^5^) with medical therapy [26]. If the mean PAP is more than 50 mmHg or the patient had a negative answer to pulmonary hypertension treatment, the mortality risk is very high, and transplantation/surgery is not allowable. In the study of Svale *et al*., the hemodynamic status and prognosis of 35 patients with PAH-PoH were evaluated [27]. The survival rates were 80, 77, and 77% at 6 months, 1, 3 years after liver transplantation. Most patients who survived for 6 months were on intravenous epoprostenol and 1/3 of them had a mean PAP less than 25 mmHg at their last visit. The authors concluded that carefully selected patients with PAH-PoH could have significant improvement in their hemodynamics with medically specific treatment after liver transplantation.

To date, we are not aware of the most appropriate specific drug to treat PAH-PoH. In many studies, PAH-PoH patients were included as a part of the study population, but typically moderate or severe liver disease (Child-Pugh B and C) was a criterion for exclusion in these trials. However, it has been clearly established that vasodilate therapy could improve the functional status (↑ functional class and 6MWT distance) and hemodynamic (↓ PVR and mean PAP, ↑ cardiac index) of PAH-PoH patients [6, 28]. Analysis of the PATENT-1 and PATENT-2 trail data demonstrated that the riociguat administration supported the same enhancement in functional class, 6 MWT distance, and PVR in PAH-PoH patients (n=13) as in other study populations [29]. In 2019, the first ever randomized controlled study of the efficacy and safety of PAH-specific therapies in patients with PAH-PoH was published [30]. Patients with PAH-PoH (n=85) with Child-Pugh class A/B and the MELD score < 19 were randomized to either the macitentan or placebo group for 12 weeks. The primary endpoint was the reduction of PVR. Macitentan has shown a decrease in PVR over 35% without improvement in functional status (WHO functional class and 6MWT distance). However, macitentan was not associated with significant hepatic side effects. The most common adverse event during the double-blind period was peripheral edema - 26% in the macitentan group *vs.* 12% in the placebo group. The post hoc analysis of this study affirmed the positive action of macitentan in the significant decreasing of the waitlist and perioperative mortality risk compared to placebo [31].

The particularity of our case is that the patient had signs of portal hypertension only in childhood. It was treated by an artificial spleno-renal shunt, which could support the blood overflow of the pulmonary artery, leading to increased wall shear stress. This patient was not monitored and was not screened for PAH until another liver disease (hepatocellular tumor) was started and aggravated. Therefore, the time of PAH onset is unknown. The hepatocellular tumor could exacerbate the vasoactive substance imbalance. At first RHC, patient had a much higher mean PAP and PVR, than PAH-PoH patients typically have. These parameters could be aggravated by bleeding, blood transfusions, and infusion therapy, which could increase the right heart load and support the clinical presentation. The vasodilated therapy was chosen to consider not its specific effects on PAH-PoH but its availability in Ukraine at that moment (sildenafil and inhalation iloprost were authorized only). However, this therapy and the successful restoration after bleeding helped to lower the mean PAP to 27 mmHg and favored the beneficial results after hepatic tumor resection. Currently, in Ukraine, there are some more available drugs for PAH treatment, including endothelin receptor blockers, which could be more convenient for patients than iloprost inhalations. But this class of drugs is heterogeneous in its negative hepatic effects. Ambrisentan or macitentan is more preferable than bosentan.

## CONCLUSION

The pathways of PAH-PoH development are still unclear. All patients, even with past history of treated portal hypertension, should be monitored closely and screened for PAH earlier for better results of treatment. The RHC should be done for PAH confirmation. The specific vasodilated therapy must be prescribed earlier, and it might improve the hemodynamic and functional status of patients with PAH-PoH. Typically, the therapy is safe and well tolerated, and it could help to provide the successful liver surgery. However, PAH-PoH patients should be managed carefully while considering the liver function and concomitant disorders. More studies are needed to better understand the aspects of specific treatment in the PAH-PoH population.

## Data Availability

The data and supportive information is available within the article.

## LIST OF ABBREVIATIONS

PAH: Pulmonary Arterial Hypertension
PCWP: Pulmonary Capillary Wedge Pressure
PVR: Pulmonary Vascular Resistance
RHC: Right Heart Catheterization

## STANDARDS OF REPORTING

CARE guidelines were followed.
